# Pre-ablation derived neutrophil-to-lymphocyte ratio is associated with time to complete disappearance after thermal ablation of benign thyroid nodules: a retrospective cohort study

**DOI:** 10.3389/fendo.2026.1817575

**Published:** 2026-05-01

**Authors:** Pei-Cheng Li, Zhong-Hua Wang, Zhen-Long Zhao, Ying Wei, Jie Wu, Wen-Jia Cai, Yan Li, Li-Li Peng, Yu-Jie Sui, Ming-An Yu

**Affiliations:** 1 Department of Thyroid Surgery, Weihai Central Hospital, Weihai, Shandong, China; 2 Department of Ultrasound Medicine, Wendeng District People’s Hospital, Weihai, Shandong, China; 3 Department of Interventional Medicine, China-Japan Friendship Hospital, Beijing, China

**Keywords:** benign thyroid nodules, complete disappearance, derived neutrophil-to-lymphocyte ratio (dNLR), microwave ablation, radiofrequency ablation, thermal ablation, volume reduction ratio (VRR)

## Abstract

**Background:**

Complete disappearance after thermal ablation for benign thyroid nodules is a time-dependent process with substantial inter-individual variability. Whether baseline systemic inflammatory–immune status contributes to the kinetics of post-ablation absorption remains unclear.

**Objective:**

To evaluate the association between pre-ablation derived neutrophil-to-lymphocyte ratio (dNLR) and time to complete disappearance (VRR = 100%) after ultrasound-guided thermal ablation of benign thyroid nodules.

**Materials and methods:**

This single-center retrospective cohort analyzed time to ultrasound-defined VRR = 100% as a time-to-event endpoint. Associations were assessed using Cox regression (log2-transformed dNLR per doubling), with stratification by ablation modality when needed. Discrimination was evaluated using Harrell’s C-index, and incremental value was assessed by comparing a prespecified clinical–procedural base model with and without dNLR.

**Results:**

Over 60 months, 95/187 (50.8%) achieved VRR = 100% (median 37 months). dNLR per doubling was associated with earlier VRR = 100% in univariable analysis (HR 2.44, 95% CI 1.63–3.65; P < 0.001) and remained independently associated in the modality-stratified multivariable model (HR 2.28, 95% CI 1.51–3.43; P < 0.001). The model including dNLR showed moderate discrimination (C-index 0.728, 95% CI 0.675–0.782) and adding dNLR modestly improved discrimination (ΔC-index ≈ 0.02; likelihood ratio test P < 0.001).

**Conclusions:**

Higher pre-ablation dNLR was independently associated with earlier complete disappearance after thermal ablation and may provide complementary information for counseling and follow-up planning; however, its incremental discrimination was modest and external validation is needed.

## 1 Introduction

Ultrasound-guided thermal ablation (radiofrequency or microwave ablation) has become an established minimally invasive option for benign thyroid nodules, achieving substantial volume reduction and symptom relief. However, nodule shrinkage and complete disappearance are time-dependent processes, and the incidence as well as the time required to reach complete disappearance (VRR = 100%) vary considerably across long-term follow-up studies (1, 2).

Previous work has mainly attributed this heterogeneity to local determinants, such as baseline volume, nodule composition, energy density, and the initial ablation ratio, all of which have been associated with post-ablation volume reduction dynamics (3–6). Nevertheless, based on our clinical observations, even among patients with comparable nodule features and similar ablation settings, marked inter-individual differences in the pace of necrotic tissue clearance and subsequent fibrotic remodeling/absorption can still be observed. This suggests that, beyond local factors, the host’s baseline systemic inflammatory–immune status may also contribute to variations in absorption speed.

The derived neutrophil-to-lymphocyte ratio (dNLR) is a readily available peripheral inflammatory index derived from routine blood counts and may reflect the relative balance between myeloid cells and non-neutrophil leukocyte populations (predominantly lymphocytes). It has been widely used as an accessible surrogate marker of systemic inflammatory–immune status and has been linked to outcomes across a range of diseases (7–9).

Accordingly, we used time to first achievement of ultrasound-defined VRR = 100% as a time-to-event endpoint to evaluate the association between pre-ablation dNLR and the rate of complete disappearance after thermal ablation for benign thyroid nodules. We further assessed the independent contribution and incremental discrimination of dNLR after accounting for key local determinants, aiming to explore whether dNLR provides complementary prognostic information for pre-procedural counseling and individualized follow-up planning.

## 2 Materials and methods

### 2.1 Study design and patient enrollment

This was a single-center retrospective cohort study. We consecutively reviewed patients with benign thyroid nodules who underwent ultrasound-guided thermal ablation (microwave ablation [MWA] or radiofrequency ablation [RFA]) at China–Japan Friendship Hospital between January 2018 and June 2020. A total of 622 patients were screened.

Inclusion criteria were as follows:

(a) benign cytology confirmed by pre-ablation ultrasound-guided fine-needle aspiration (FNA), with no highly suspicious malignant features on pre-ablation ultrasound;
(b) only one target nodule was selected for each patient;
(c) ablation indications, including compressive symptoms, cosmetic concerns, nodule growth on follow-up, or a clear preference for intervention;
(d) complete baseline data, including pre-ablation ultrasound assessment and clinical data such as complete blood count (CBC) obtained within 2 days before ablation;
(e) available post-ablation ultrasound follow-up records with corresponding time points for outcome assessment.

Exclusion criteria were as follows:

(a) malignant or indeterminate cytology on FNA;
(b) non-thermal ablation therapy (e.g., ethanol ablation);
(c) history of thyroid surgery or prior thyroid ablation;
(d) missing key baseline or follow-up data;
(e) incomplete ablation;
(f) any infection within 2 weeks before ablation or a history of hematologic disease.

According to these criteria, 435 patients were excluded, and 187 patients were included in the final analysis.

The study protocol was approved by the Ethics Committee of China–Japan Friendship Hospital. Given the retrospective nature of the study and the use of de-identified clinical data, the requirement for informed consent was waived.

### 2.2 Pre-ablation clinical and laboratory assessment

All patients underwent routine pre-procedural evaluation and ultrasound examination. Baseline demographic and clinical information (e.g., age, sex, and medical history), laboratory parameters (complete blood count and thyroid function tests), conventional ultrasound features (e.g., location, margin, composition, echogenicity, calcification, and honeycomb change), and contrast-enhanced ultrasound (CEUS) characteristics were collected. For CEUS, perflubutane microbubbles (Sonazoid) were used as the contrast agent.

The derived neutrophil-to-lymphocyte ratio (dNLR) was calculated as: dNLR = NEUT/(WBC − NEUT), where NEUT denotes the absolute neutrophil count and WBC denotes the total white blood cell count (7).

Nodule volume was calculated using the ellipsoid formula: V (cm³) = (π/6) × a × b × c, where a, b, and c represent the three orthogonal diameters measured in centimeters (10).

Energy density was defined as total delivered energy divided by baseline nodule volume and was expressed as J/mm³ (baseline volume was converted from cm³ to mm³ for calculation) (11).

### 2.3 Ablation procedure

All ablations were performed by experienced physicians (attending level or above) under local anesthesia with real-time ultrasound guidance. Ultrasound examinations were performed using ultrasound systems from GE Healthcare (General Electric Company, USA). For MWA, the HYH-50C microwave ablation system (Nanjing ECO Microwave System) or the KY-2000 system was used; for RFA, the Cool-tip Radiofrequency Ablation System (Covidien) was used.

Under continuous ultrasound monitoring, the antenna/electrode was inserted into the target nodule. Hydrodissection was routinely performed to protect critical surrounding structures; 0.9% saline was used for MWA and sterile water for injection was used for RFA. Ablation was performed in a stepwise manner using a moving-shot/multiple overlapping technique based on nodule size, location, and shape, until the ablation zone demonstrated a typical hyperechoic change covering the target region. Before completion, CEUS was performed to assess the ablation extent; additional ablation was applied if residual enhancing foci were detected, until complete ablation was achieved.

After the procedure, patients were observed in the recovery area for approximately 30 minutes. Local cold compression was applied, and no peri-procedural anti-inflammatory drugs were administered.

### 2.4 Follow-up schedule and outcome definitions

Patients were scheduled for follow-up at 1, 3, 6, 9, and 12 months after ablation and every 6 months thereafter. At each visit, the ablation zone volume and volume reduction ratio (VRR) were recorded. VRR was calculated as: VRR (%) = (V_0_ − V_t_)/V_0_ × 100%, where V_0_ is baseline volume and V_t_ is the volume at each follow-up (12).

Complete disappearance (VRR = 100%) was defined as no identifiable residual lesion on ultrasound. Thus, the primary endpoint was an ultrasound-defined imaging endpoint rather than a pathology-confirmed endpoint. Time to complete disappearance was defined as the first follow-up visit at which VRR reached 100%. Patients who did not reach VRR = 100% by the end of follow-up were censored at the last follow-up visit.

### 2.5 Statistical analysis

All statistical analyses were performed in R (R Foundation for Statistical Computing) and executed in RStudio (Posit Software, PBC; version 2025.09.2 + 418). Continuous variables were summarized as mean ± standard deviation or median (interquartile range), as appropriate, and categorical variables as number (percentage). For descriptive comparisons between groups, continuous variables were compared using Student’s t test or the Mann–Whitney U test, and categorical variables using the χ² test or Fisher’s exact test, as appropriate.

Time to complete disappearance (VRR = 100%) was analyzed as a time-to-event outcome: the first ultrasound assessment documenting VRR = 100% was treated as the event, and patients without the event were censored at the last follow-up. The overall cumulative incidence of complete disappearance was visualized as 1 − Kaplan–Meier (KM). Univariable and multivariable Cox proportional hazards models were fitted to estimate hazard ratios (HRs) with 95% confidence intervals (CIs), with ties handled using the Efron method. Skewed continuous variables were log2-transformed and interpreted per doubling. The primary multivariable model was a prespecified parsimonious clinical–procedural model including age, sex, baseline nodule volume, composition, energy density, and Hashimoto’s thyroiditis. Ablation modality was handled by stratification when the proportional hazards assumption was violated, as assessed using Schoenfeld residuals. To reduce overfitting and preserve model stability after stratification, variables that were significant only in univariable analyses (e.g., echogenicity, honeycomb sign, CEUS pattern, and calcification) were not forced into the primary multivariable model and were interpreted as exploratory findings. Patients with missing key baseline CBC data or key follow-up ultrasound data were excluded according to the prespecified eligibility criteria; therefore, the primary analysis was effectively a complete-case analysis, and no imputation was performed. Model discrimination was assessed using Harrell’s C-index. To quantify the incremental value of dNLR, discrimination was compared between a predefined clinical–procedural base model (excluding dNLR) and the base model plus dNLR. Nested models were additionally compared using the likelihood ratio test. All tests were two-sided, and P < 0.05 was considered statistically significant.

## 3 Results

### 3.1 Study population and overall baseline characteristics

We retrospectively reviewed 622 patients who underwent microwave ablation (MWA) or radiofrequency ablation (RFA) for benign thyroid nodules between January 2018 and June 2020. After excluding 435 patients who did not meet the inclusion criteria, 187 patients were finally included in the analysis. The median age was 48.00 (37.00–59.00) years; 47 patients (25.1%) were male and 140 (74.9%) were female. The median baseline nodule volume was 6.16 (2.10–16.02) cm³. The median energy density was 1.66 (1.07–2.64) J/mm³, and the median ablation time and power were 217.00 (115.00–353.00) s and 30.00 (30.00–60.00) W, respectively. Within the maximum follow-up window of 60 months, 95/187 (50.8%) patients achieved the endpoint of complete disappearance (VRR = 100%) (**Table 1**).

**Table 1:** Baseline characteristics of the study cohort.

Characteristics	Overall (n = 187)
Age (years)	48.00 (37.00–59.00)
Sex	
Male	47 (25.1%)
Female	140 (74.9%)
dNLR	1.61 (1.30–2.08)
Initial nodule volume (cm³)	6.16 (2.10–16.02)
Nodule location	
Right lobe	94 (50.3%)
Left lobe	87 (46.5%)
Isthmus	6 (3.2%)
Composition	
Cystic	24 (12.8%)
Mixed (cystic–solid)	48 (25.7%)
Solid	115 (61.5%)
Echogenicity	
Anechoic	17 (9.1%)
Hyperechoic	16 (8.6%)
Isoechoic	66 (35.3%)
Hypoechoic	88 (47.1%)
Calcification	
Yes	26 (13.9%)
No	161 (86.1%)
CEUS pattern	
Hyperenhancement	160 (85.6%)
Non-hyperenhancement	27 (14.4%)
Ablation type	
Microwave ablation (MWA)	109 (58.3%)
Radiofrequency ablation (RFA)	78 (41.7%)
Energy density (J/mm³)	1.66 (1.07–2.64)
Ablation time (s)	217.00 (115.00–353.00)
Ablation power (W)	30.00 (30.00–60.00)
Follow-up to event/last visit (months)	29.00 (24.00–35.00)
Complete disappearance (VRR = 100%)	95/187 (50.8%)
Hashimoto’s thyroiditis	
No	163 (87.2%)
Yes	24 (12.8%)
Hypertension	
No	145 (77.5%)
Yes	42 (22.5%)
Diabetes	
No	166 (88.8%)
Yes	21 (11.2%)
Smoking	
No	166 (88.8%)
Yes	21 (11.2%)
Alcohol consumption	
No	177 (94.7%)
Yes	10 (5.3%)

### 3.2 Overall cumulative incidence of complete disappearance (1 − Kaplan–Meier)

**Figure 1:**
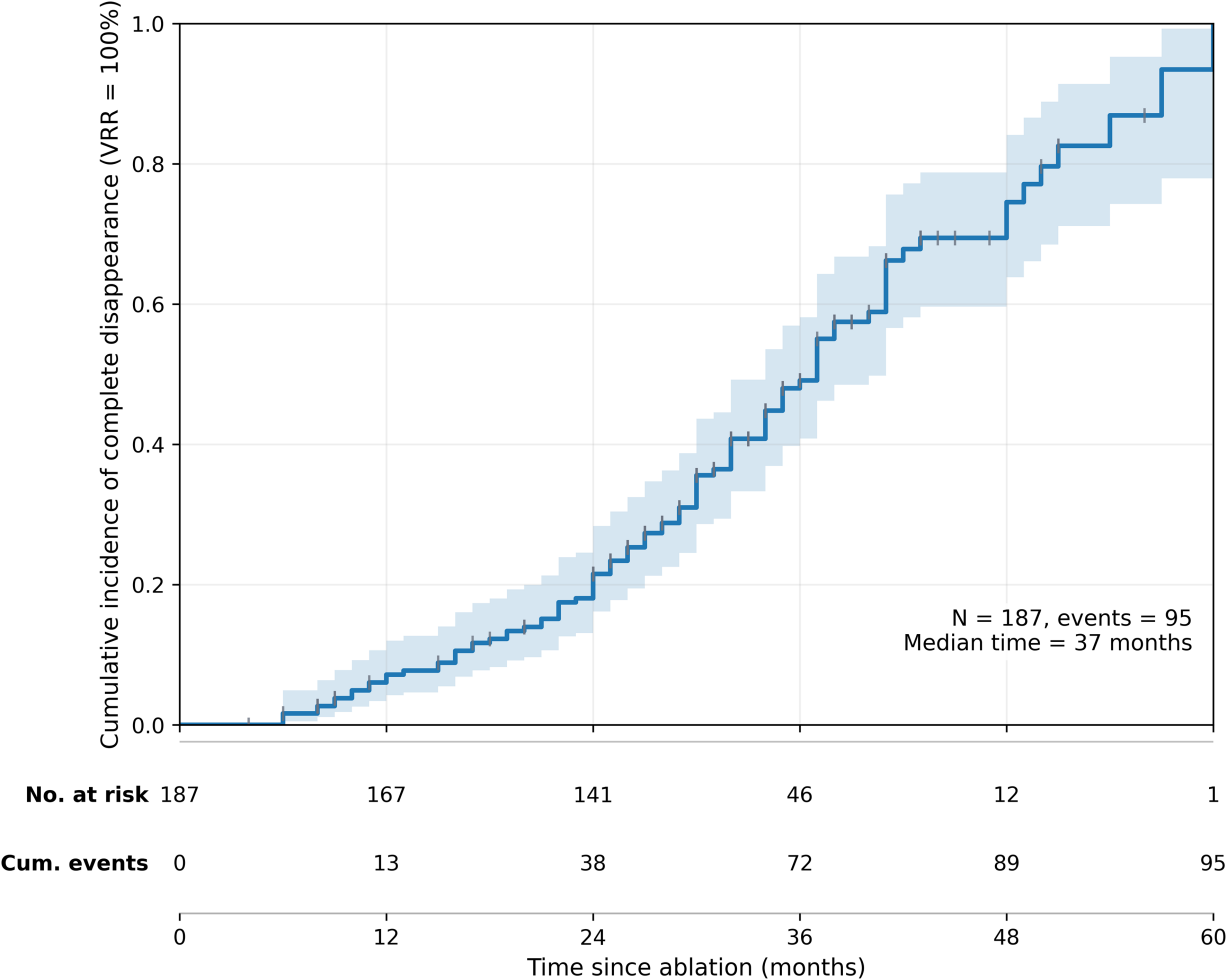
Overall cumulative incidence of complete disappearance (VRR = 100%) after ultrasound-guided thermal ablation of benign thyroid nodules, estimated as 1 − Kaplan–Meier. The solid line indicates the estimated cumulative incidence and the shaded area indicates the 95% confidence interval. Vertical tick marks indicate censoring. The lower panel shows the number at risk and cumulative number of events at 0, 12, 24, 36, 48, and 60 months. N = 187; events = 95; Kaplan–Meier–estimated median time to complete disappearance = 37 months.

During follow-up, the overall cumulative incidence of complete disappearance (VRR = 100%) increased progressively over time. As shown in **Figure 1**, the estimated cumulative incidence (1 − Kaplan–Meier) was 7.2% at 12 months, 21.5% at 24 months, 49.1% at 36 months, and 74.5% at 48 months. The Kaplan–Meier–estimated median time to complete disappearance was 37 months.

### 3.3 Baseline characteristics by dNLR group

For descriptive purposes, patients were stratified by the median pre-ablation dNLR (1.61) into a high-dNLR group (n = 93) and a low-dNLR group (n = 94). Baseline nodule characteristics (volume, location, composition, echogenicity, calcification, honeycomb sign, and CEUS pattern) and treatment-related parameters (ablation modality, ablation duration, power, and energy density) were comparable between groups, as were Hashimoto’s thyroiditis, comorbidities (hypertension and diabetes), and lifestyle factors (smoking and alcohol consumption) (all P > 0.05). Patients in the high-dNLR group were slightly older than those in the low-dNLR group (median 50.0 [IQR 40.0–60.0] vs 45.0 [IQR 36.0–57.0] years; P = 0.036) (**Table 2**).

**Table 2:** Baseline characteristics stratified by median pre-ablation dNLR.

Characteristics	Overall (n = 187)	High dNLR (n = 93)	Low dNLR (n = 94)	P value
Age (years)	48.00 (37.00–59.00)	50.00 (40.00–60.00)	45.00 (36.00–57.00)	0.036
Sex				0.527
Male	47 (25.1%)	21 (22.6%)	26 (27.7%)	
Female	140 (74.9%)	72 (77.4%)	68 (72.3%)	
Initial nodule volume (cm³)	6.16 (2.10–16.02)	6.16 (2.26–17.30)	6.09 (1.82–15.81)	0.857
Nodule location				0.997
Right lobe	94 (50.3%)	47 (50.5%)	47 (50.0%)	
Left lobe	87 (46.5%)	43 (46.2%)	44 (46.8%)	
Isthmus	6 (3.2%)	3 (3.2%)	3 (3.2%)	
Composition				0.386
Cystic	24 (12.8%)	15 (16.1%)	9 (9.6%)	
Mixed (cystic–solid)	48 (25.7%)	22 (23.7%)	26 (27.7%)	
Solid	115 (61.5%)	56 (60.2%)	59 (62.8%)	
Echogenicity				0.099
Anechoic	17 (9.1%)	13 (14.0%)	4 (4.3%)	
Hyperechoic	16 (8.6%)	8 (8.6%)	8 (8.5%)	
Isoechoic	66 (35.3%)	28 (30.1%)	38 (40.4%)	
Hypoechoic	88 (47.1%)	44 (47.3%)	44 (46.8%)	
Calcification				0.147
Yes	26 (13.9%)	9 (9.7%)	17 (18.1%)	
No	161 (86.1%)	84 (90.3%)	77 (81.9%)	
CEUS pattern				0.223
Hyperenhancement	160 (85.6%)	83 (89.2%)	77 (81.9%)	
Non-hyperenhancement	27 (14.4%)	10 (10.8%)	17 (18.1%)	
Ablation type				0.834
Microwave ablation (MWA)	109 (58.3%)	53 (57.0%)	56 (59.6%)	
Radiofrequency ablation (RFA)	78 (41.7%)	40 (43.0%)	38 (40.4%)	
Energy density (J/mm³)	1.66 (1.07–2.64)	1.55 (1.07–2.57)	1.79 (1.08–2.99)	0.252
Ablation time (s)	217.00 (115.00–353.00)	206.00 (120.00–350.00)	222.50 (113.25–353.25)	0.609
Ablation power (W)	30.00 (30.00–60.00)	30.00 (30.00–60.00)	30.00 (30.00–60.00)	0.668
Hashimoto’s thyroiditis				> 0.999
No	163 (87.2%)	81 (87.1%)	82 (87.2%)	
Yes	24 (12.8%)	12 (12.9%)	12 (12.8%)	
Hypertension				0.206
No	145 (77.5%)	68 (73.1%)	77 (81.9%)	
Yes	42 (22.5%)	25 (26.9%)	17 (18.1%)	
Diabetes				0.625
No	166 (88.8%)	81 (87.1%)	85 (90.4%)	
Yes	21 (11.2%)	12 (12.9%)	9 (9.6%)	
Smoking				0.368
No	166 (88.8%)	85 (91.4%)	81 (86.2%)	
Yes	21 (11.2%)	8 (8.6%)	13 (13.8%)	
Alcohol consumption				0.330
No	177 (94.7%)	90 (96.8%)	87 (92.6%)	
Yes	10 (5.3%)	3 (3.2%)	7 (7.4%)	

### 3.4 Univariable cox regression

**Figure 2:**
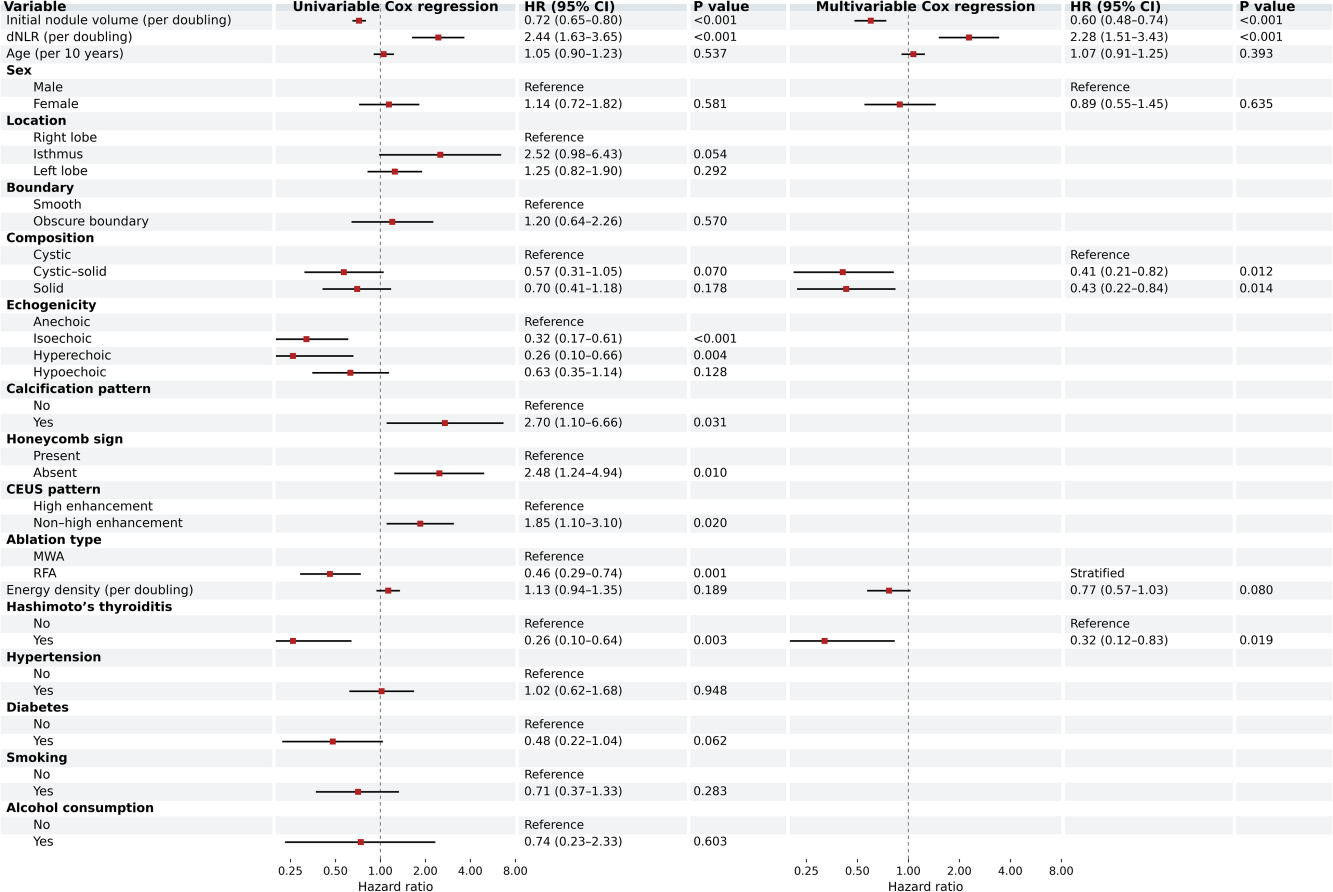
Forest plot of factors associated with time to complete disappearance (VRR = 100%) in univariable Cox regression and modality-stratified multivariable Cox regression analyses. Points indicate hazard ratios, and horizontal bars indicate 95% confidence intervals. Hazard ratios >1 indicate a higher hazard of achieving VRR = 100% (i.e., a shorter time to complete disappearance).

In univariable Cox models, pre-ablation dNLR (per doubling) was associated with a higher rate of achieving VRR = 100% (HR = 2.44, 95% CI 1.63–3.65; P < 0.001), whereas baseline nodule volume (per doubling) was inversely associated with the endpoint (HR = 0.72, 95% CI 0.65–0.80; P < 0.001). Echogenicity, ablation modality, Hashimoto’s thyroiditis, honeycomb sign, CEUS pattern, and calcification pattern were also associated with time to complete disappearance at the overall level (all global P < 0.05) (**Figure 2**).

### 3.5 Multivariable cox regression (stratified by ablation modality)

Given evidence of non-proportional hazards for ablation modality, the primary multivariable analysis was performed using a Cox model stratified by ablation modality. After adjustment for age, sex, baseline nodule volume, composition, energy density, and Hashimoto’s thyroiditis, dNLR (per doubling) remained independently associated with a higher hazard of complete disappearance (HR = 2.28, 95% CI 1.51–3.43; P < 0.001). Baseline nodule volume (per doubling) remained inversely associated with the endpoint (HR = 0.60, 95% CI 0.48–0.74; P < 0.001). Compared with cystic nodules, mixed (cystic–solid) and solid nodules showed lower rates of reaching VRR = 100% (HR = 0.41, 95% CI 0.21–0.82; P = 0.012; and HR = 0.43, 95% CI 0.22–0.84; P = 0.014, respectively). Hashimoto’s thyroiditis was also associated with a lower hazard of complete disappearance (HR = 0.32, 95% CI 0.12–0.83; P = 0.019), while energy density showed only a borderline association (HR = 0.77, 95% CI 0.57–1.03; P = 0.080). Age and sex were not significantly associated with time to VRR = 100% in the adjusted model (**Figure 2**).

### 3.6 Model discrimination

Harrell’s C-index was calculated within ablation modality strata. The clinical–procedural base model (age, sex, baseline volume, composition, energy density, and Hashimoto’s thyroiditis; stratified by ablation modality) showed moderate discrimination (C-index = 0.708). After adding dNLR (log2-transformed), discrimination improved to C-index = 0.728 (ΔC-index = 0.020), and model fit improved (likelihood ratio χ² = 15.67; P < 0.001). A model including dNLR alone showed limited discrimination (C-index = 0.580).

## 4 Discussion

This retrospective cohort study examined whether the pre-ablation derived neutrophil-to-lymphocyte ratio (dNLR) is associated with the time to complete disappearance (VRR = 100%) following ultrasound-guided thermal ablation of benign thyroid nodules. Among 187 patients treated with microwave or radiofrequency ablation, a higher pre-ablation dNLR was associated with an earlier achievement of VRR = 100% (i.e., a shorter time to complete disappearance). After adjustment for age, sex, baseline nodule volume, composition, energy density, and Hashimoto’s thyroiditis, and with stratification by ablation modality to address non-proportional hazards, dNLR (per doubling) remained independently associated with complete disappearance (HR = 2.28, 95% CI 1.51–3.43). The multivariable model including dNLR showed moderate internal discrimination (Harrell’s C-index = 0.728, 95% CI 0.675–0.782). From a prediction standpoint, dNLR alone showed limited discrimination (C-index = 0.580), and adding dNLR to the clinical–procedural base model provided a modest improvement in discrimination (ΔC-index = 0.020) and improved model fit (likelihood ratio test P < 0.001), suggesting complementary prognostic information beyond conventional nodule- and treatment-related factors rather than a standalone clinically actionable prediction tool.

From an immunological perspective, dNLR is a pragmatic index derived from routine blood counts that may reflect the relative predominance of neutrophil-associated innate immune activity over lymphocyte-associated adaptive immune components (13). Prior studies have commonly used dNLR or NLR as readily accessible surrogates of systemic inflammatory burden and have demonstrated their prognostic relevance across a broad range of malignancies and inflammation-related conditions (7, 13). However, accumulating evidence in recent years indicates that neutrophils are not merely mediators of tissue injury or adverse outcomes; rather, they may exert context-dependent functions after sterile injury, including coordinating inflammatory resolution, facilitating necrotic debris clearance, and contributing to tissue repair and remodeling processes (14–17).

Thermal ablation induces sterile coagulative necrosis within the target lesion, and subsequent volume reduction largely depends on the degradation of necrotic tissue, resolution of inflammation, and the ensuing fibrotic remodeling and contraction. Experimental evidence suggests that, in the early phase of injury, neutrophils contribute to the breakdown of necrotic tissue by releasing proteases and matrix metalloproteinases (MMPs) (15, 18). Other studies have further suggested that neutrophils may facilitate the transition from inflammatory initiation to resolution by releasing proteases (including MMP-9) that can degrade pro-inflammatory mediators and selected damage-associated molecular patterns (DAMPs), thereby helping to limit excessive leukocyte recruitment and promote inflammatory resolution (16, 17, 19). In light of the observed association (approximately a 2.3-fold higher hazard of achieving VRR = 100% per doubling of dNLR), a biologically plausible interpretation is that a higher pre-ablation dNLR may reflect a systemic milieu permissive of more rapid early myeloid engagement and debris clearance after ablation, which could in turn favor an earlier transition from acute inflammation to absorption/remodeling and a shorter time to complete disappearance.

In our stratified multivariable Cox model, in addition to dNLR, baseline nodule volume, nodule composition, and Hashimoto’s thyroiditis were also independently associated with time to complete disappearance. First, each doubling of baseline nodule volume was associated with an approximately 40% lower hazard of achieving VRR = 100% (HR = 0.60), which is consistent with large cohort studies reporting that larger baseline volume predicts a lower likelihood of treatment success and/or a slower improvement in VRR over time (11, 12). Mechanistically, a larger baseline volume likely translates into a greater burden of coagulative necrosis and a longer trajectory of debris clearance and repair. In addition, absorption kinetics may also depend on the energy delivered per unit volume; for larger nodules, insufficient energy density may result in a lower initial effective ablation ratio, thereby prolonging the time required to reach complete disappearance (11, 20).

Second, nodule composition also emerged as an important determinant. Compared with cystic nodules, mixed cystic–solid and solid nodules were associated with a lower hazard of complete disappearance, in line with previous reports (21, 22). This may reflect faster early collapse of cystic components after fluid evacuation, whereas predominantly solid tissue generally requires a longer period for degradation and remodeling.

Finally, Hashimoto’s thyroiditis was a negative factor for VRR in our cohort, consistent with several retrospective studies showing that patients with Hashimoto’s thyroiditis require a longer time to achieve substantial volume reduction or complete disappearance after ablation (23, 24). Notably, given our finding that pre-ablation dNLR was closely associated with absorption speed, a disease state dominated by lymphocytic infiltration—such as Hashimoto’s thyroiditis—may, at least in part, interact with the systemic inflammatory–immune balance captured by dNLR by shifting local inflammatory–immune homeostasis, thereby jointly shaping post-ablation resorption and remodeling and contributing to inter-individual variability in absorption kinetics. However, because thyroid autoantibody data (e.g., TPOAb and TgAb) were not systematically available in this retrospective cohort, we were unable to formally test whether autoantibody status modified the association between dNLR and time to complete disappearance.

From a clinical perspective, most prior studies on the efficacy of thermal ablation for benign thyroid nodules have emphasized local determinants—including baseline nodule volume, compositional characteristics, and ablation energy parameters—as key drivers of post-ablation volume reduction (25, 26). However, existing work has predominantly focused on nodule-specific features and procedural parameters, with comparatively less attention to inter-individual differences in baseline systemic inflammatory–immune status that could influence post-ablation absorption kinetics. In the present study, we used time to VRR = 100% as the endpoint and applied Cox regression to evaluate factors associated with absorption speed. Notably, we incorporated the pre-ablation peripheral inflammatory index dNLR as a clinically available marker of systemic inflammatory–immune balance. Our results showed that, even after accounting for conventional imaging characteristics and ablation-related parameters, dNLR remained associated with absorption speed (HR = 2.28), suggesting that complete disappearance may be shaped not only by local lesion characteristics but also by host-level inflammatory–immune milieu. Collectively, these findings add a complementary immunological perspective to the interpretation of post-ablation resorption dynamics and may help frame patient counseling and follow-up planning; however, the incremental discrimination gain was modest and the findings should be interpreted as hypothesis-generating until externally validated.

The study has several limitations. First, this was a single-center retrospective analysis. Although we adjusted for potential confounders using multivariable and stratified Cox regression models, selection bias and residual/unmeasured confounding cannot be completely excluded. Second, complete disappearance (VRR = 100%) was an ultrasound-defined imaging endpoint rather than a pathology-confirmed endpoint. In benign thyroid nodules successfully treated by thermal ablation, surgery is usually not subsequently performed, making systematic pathological confirmation impractical. Third, several potentially relevant biological or treatment-related factors were not systematically captured. dNLR may be influenced by smoking status, obesity, and metabolic conditions, and thyroid autoantibodies were not comprehensively available, precluding a formal assessment of interactions between TPOAb/TgAb and dNLR. In our center, no peri-procedural anti-inflammatory drugs were administered in this cohort. However, peri-procedural glucocorticoids and other treatment-related factors that could affect inflammatory resolution and absorption kinetics were not systematically recorded and therefore could not be included in the adjusted analyses. Future studies incorporating thyroid autoantibodies, C-reactive protein (CRP), erythrocyte sedimentation rate (ESR), metabolic markers, standardized treatment-exposure data, and postoperative longitudinal blood count dynamics are warranted to further validate and refine the mechanistic interpretation of this association. Finally, the incremental discrimination gain from adding dNLR was modest, and no external validation cohort was available; therefore, the predictive value of dNLR should be interpreted cautiously until confirmed in prospective, multicenter studies.

In conclusion, a higher pre-ablation dNLR was independently associated with an earlier complete disappearance (VRR = 100%) after thermal ablation of benign thyroid nodules. These findings suggest that baseline systemic inflammatory–immune status may be a clinically relevant correlate of post-ablation resorption dynamics. As a simple and readily available pre-ablation biomarker, dNLR may provide complementary information for patient counseling regarding expected absorption trajectories and individualized follow-up planning, but its incremental discrimination was modest and external validation is needed before broader clinical application.

## Data Availability

The raw data supporting the conclusions of this article will be made available by the authors, without undue reservation.
