# Correction: Pre-ablation derived neutrophil-to-lymphocyte ratio is associated with time to complete disappearance after thermal ablation of benign thyroid nodules: a retrospective cohort study

**DOI:** 10.3389/fendo.2026.1921835

**Published:** 2026-07-09

**Authors:** Pei-Cheng Li, Zhong-Hua Wang, Zhen-Long Zhao, Ying Wei, Jie Wu, Wen-Jia Cai, Yan Li, Li-Li Peng, Yu-Jie Sui, Ming-An Yu

**Affiliations:** 1 Department of Thyroid Surgery, Weihai Central Hospital, Weihai, Shandong, China; 2 Department of Ultrasound Medicine, Wendeng District People’s Hospital, Weihai, Shandong, China; 3 Department of Interventional Medicine, China-Japan Friendship Hospital, Beijing, China

**Keywords:** benign thyroid nodules, complete disappearance, derived neutrophil-to-lymphocyte ratio (dNLR), microwave ablation, radiofrequency ablation, thermal ablation, volume reduction ratio (VRR)

In the published article, there was an error in the **Funding** statement. The statement was erroneously given as:

“The author(s) declared that financial support was not received for this work and/or its publication.”

The funder National High Level Hospital Clinical Research Funding (2025-NHLHCRF-JBGS-A-WZ-01), awarded to Ming-An Yu, was erroneously omitted.

The correct Funding statement is:

“The author(s) declared that financial support was received for the research and publication of this article. This work, including the article processing charge (APC), was supported by the National High Level Hospital Clinical Research Funding (2025-NHLHCRF-JBGS-A-WZ-01) to Ming-An Yu. The funder had no role in the study design, data collection and analysis, decision to publish, or preparation of the manuscript.”

The original version of this article has been updated.

